# Increased Kynurenine Indicates a Fatal Course of COVID-19

**DOI:** 10.3390/antiox10121960

**Published:** 2021-12-07

**Authors:** Harald Mangge, Markus Herrmann, Andreas Meinitzer, Sabine Pailer, Pero Curcic, Zdenka Sloup, Magdalena Holter, Florian Prüller

**Affiliations:** 1 Clinical Institute of Medical and Chemical Laboratory Diagnostics, Medical University of Graz, 8036 Graz, Austria; markus.herrmann@medunigraz.at (M.H.); andreas.meinitzer@medunigraz.at (A.M.); sabine.pailer@medunigraz.at (S.P.); pero.curcic@medunigraz.at (P.C.); zdenka.sloup@medunigraz.at (Z.S.); florian.prueller@medunigraz.at (F.P.); 2 Institute of Medical Informatics, Statistics and Documentation, Medical University of Graz, 8036 Graz, Austria; magdalena.holter@medunigraz.at

**Keywords:** kynurenine, tryptophan, COVID-19

## Abstract

**(1) Background**: An inefficient immune response accompanied by an overwhelming inflammatory reaction is involved in severe courses of COVID-19. Kynurenine (KYN) has important immune-modulatory functions and may contribute to a failure in controlling SARS-CoV-2. The present study aims to explore biomarkers that hint at a fatal outcome of COVID-19 early on. **(2) Methods**: We established a cohort of 148 hospitalized COVID-19 patients for this study. Thirty-one patients died due to a severe COVID-19 course, and 117 recovered within 90 days. We built a biobank by collecting left-over material from these patients whenever blood arrived at the central laboratory of our University hospital for analysis of routine markers. The scientific laboratory analysis comprised KYN, Tryptophan (TRP), KYN/TRP ratio, ferritin, interleukin-6 (IL-6), C-reactive protein (CRP), creatinine, N-terminal pro-natriuretic peptide (NTproBNP), troponin T (TnT), fibrinogen, D-Dimer, prothrombin time (PT), activated partial thromboplastin time (aPTT), antithrombin (AT), protein C, protein S, factor XIII, lupus aPTT, angiotensin-2, vitamin D metabolites, and telomeres in all COVID-19 patients. Basic clinical characteristics and anteceding diseases including cardiovascular, oncologic, renal, hypertension, pulmonary, metabolic (diabetes, obesity) were recorded in a database together with the laboratory data. **(3) Results**: At the time of diagnosis of SARS-CoV-2 infection those patients who deceased within 90 days afterwards due to COVID-19, had a significantly higher age, higher KYN, KYN/TRP ratio, ferritin, creatinine, and NTproBNP values than SARS-CoV-2 patients who survived COVID-19 along the same time span. In a Kaplan-Meier analysis the variables age, KYN, ferritin, D-Dimer, TnT, NTproBNP, and creatinine showed a significant influence on survival time. Gender, however, showed no influence. In a combined Cox regression analysis KYN had the highest hazard ratio (1.188, 95% CI: 1.071–1.319) followed by age (1.041, 95% CI: 1.011–1.073). In a ROC analysis, KYN values above the cut off limit of 4.82 nmol/l (as specified by Youden index) had a sensitivity of 82% (95% CI: 66–95%) and a specificity of 72% (95% CI: 65–82%) to predict COVID-19 related death within 90 days observation time. **(4) Conclusions**: Kynurenine is a promising blood biomarker to predict an increased risk of mortality in SARS-CoV-2 infected people already at the time of the first positive SARS-CoV-2 verification detected in these persons.

## 1. Introduction

Kynurenine (KYN) is a metabolite of the amino acid tryptophan (TRP) used in the production of niacin [1,2,3,4]. Kynurenine is synthesized by the enzymes TRP 2,3-dioxygenase (TDO), originating mainly from the liver [2], indoleamine 2,3-dioxygenase-1 (IDO1) which is made in many tissues in response to immune activation [3,4], and the much less studied IDO2 expressed in the liver, kidneys, and antigen presenting cells [4]. The role of IDO2 in immune function is still being determined [4]. Kynurenine and its further breakdown products carry out important biological functions, including regulation of the immune response [5,6], involvement in oxidative stress [7], and dilating blood vessels during inflammation [8]. Some cancers increase KYN production, which promotes tumor growth [3]. Evidence suggests that increased KYN production is also involved in neurologic/psychiatric diseases [9,10,11], Alzheimer’s disease [12], obesity [2], and cardiovascular disease (CVD) [13]. Dysfunctional states of distinct steps of the KYN pathway (e.g., kynurenic acid, quinolinic acid, anthranilic acid, 3-hydroxykynurenine) have been related to a number of disorders [11,14,15,16].

Most tryptophan degraded by the kynurenine pathway is fully oxidized to CO2 [17], with NAD being a relatively minor side product [1]. NAD exists as NAD^+^, an oxidizing agent, and in a reduced form as NADH (H for hydrogen) [1]. Thus, endogenous TRP and its metabolites can interact and/or produce reactive oxygen species in tissues and cells [1]. This is of great importance for oxidative stress where alterations in KYN pathway metabolites interact with energetic deficit, cell death, and inflammatory events [1].

Moreover, the KYN pathway has received increasing attention concerning infectious disease as its connection to inflammation, the immune system, oxidative stress, and neurological conditions became apparent [7,18,19]. Evidence exists, that the KYN pathway is also involved in severe acute respiratory syndrome coronavirus 2 (SARS-CoV-2) [18,19]. Herein, an altered TRP metabolism may disturb an effective immune response, involved in oxidative stress generation [20], and promote long-term symptoms in survivors of coronavirus disease 2019 (COVID-19) [21] including post COVID depression [22].

Lawler et al. demonstrated that nine metabolites were elevated in plasma of SARS-CoV-2 patients (i.e., quinolinic acid, glutamic acid, nicotinic acid, aspartic acid, neopterin, KYN, phenylalanine, 3-OH-KYN, and taurine); while four metabolites were lower (TRP, histidine, indole-3-acetic acid, and citrulline) [23]. Accordingly, recent metabolomics analysis of COVID-19 patients identified anthranilic acid, a metabolite of the KYN pathway as a poor prognostic biomarker, correlating with the maintenance of high interleukin (IL)-10 and IL-18 [24]. As products of the KYN pathway, KYN or anthranilic acid have immunosuppressive properties, and their therapeutic utility to inhibit IDO and TPO was discussed for patients with severe COVID-19 [24].

These data prompted us to investigate herein KYN, the master marker of TRP degradation, for its potential to predict a fatal outcome in a cohort of hospitalized COVID-19 patients.

## 2. Materials and Methods

### 2.1. Study Design

We established a biobank (Alpe_Adria_Coronavirus_Cohort, ALDOCOV) by collecting leftovers of blood samples from patients suffering from COVID-19 whenever sent to the central laboratory of our University hospital between April and December 2020. After the completion of all routine laboratory testing, residual material was stored at −80 °C until batched analysis. In this retrospective study, we measured the plasma concentrations of KYN and TRP by a validated LC-UV/Fluorometric detection method in blood samples of the first visit of 148 COVID-19 patients. Besides KYN and TRP the laboratory analysis comprised ferritin, interleukin-6 (IL-6), C-reactive protein (CRP), creatinine, N-terminal-pro hormone B-type pro-natriuretic peptide (NTproBNP), troponin T (TnT), and D-Dimer, prothrombin time (PT), activated partial thromboplastin time (aPTT), antithrombin (AT), protein C, protein S, and factor XIII. Basic clinical characteristics and anteceding diseases including cardiovascular, oncologic, renal, hypertension, pulmonary, and metabolic (diabetes, obesity) were recorded in the database. Anthropometric and clinical data, as well as outcome data were obtained from the laboratory and hospital information systems. The primary outcome was death within 90 days after admission. Respiratory support with oxygen was used as the secondary endpoint. The institutional ethics committee of the Medical University of Graz (EK 32-475 ex 19/20) approved this study.

### 2.2. Laboratory Analysis

The diagnosis of COVID-19 was confirmed in all patients with viral reverse transcriptase PCR using the Xpert^®^ Xpress SARS-CoV-2 (singleplex) cartridge and device (GeneXpert, Cepheid GmbH, 47,807 Krefeld, Germany), as previously described [25,26]. Interleukin-6 (IL-6), ferritin, NTproBNP, cTnT, and C-reactive protein (CRP) were measured with commercial immunoassays on a COBAS 8000 analyzer (Roche Diagnostics, Rotkreuz, Switzerland). Kynurenine and TRP were measured by high performance liquid chromatography with a simultaneous ultraviolet and fluorometric detection system [27]. In brief, 100 µL plasma sample was deproteinized by adding 100 µL of 5% (*v/v*) perchloric acid. After vortexing and 5 min centrifugation at 11,000× *g*, 20 μL of the clear supernatant was injected into the chromatographic system. Separations were done on a Chromolith RP18e column (100 × 4.6 mm, 5µm, Merck, Darmstadt, Germany) at 30 °C by isocratic elution with a mobile phase (pH 4.9) consisting of 50 mmol/L ammonium acetate, 250 mmol/L zinc acetate and 3% (*v/v*) acetonitrile, at a flow-rate of 0.8 mL/min. Tryptophan and KYN were detected on an Agilent 1200 VWD detector (Agilent, Palo Alto, CA, USA) at 235 nm; KYN was detected fluorometrically on an Agilent 1260 FLD detector. The acquisition and processing of the chromatograms used an Agilent 1200 system equipped with a Chemstation software (Agilent, Palo Alto, CA, USA). All reagents were p.A. grade from Merck (Darmstadt, Germany). The intra-assay CVs for different concentrations varied between 0.7–2.9% for TRP, 1.7–4.3% for KYN, and 2.6–4.5% for KYNA. The inter-assay CVs for TRP, KYN, and KYNA ranged between 6.3–9%, 2.0–5.4%, and 8.4–11.6%, respectively [28]. D-Dimer, prothrombin time, activated partial thromboplastin time (aPTT), antithrombin (AT), protein C, protein S, and factor XIII were analyzed on a Siemens Atellica COAG-360 analyzer (Siemens Healthineers, Marburg, Germany).

### 2.3. Data Analysis

For descriptive statistics, median, minimum, and maximum were determined for continuous not normally distributed variables. Categorical outcome variables were displayed as absolute and relative frequencies and differences were assessed either by a Chi^2^ or by a Fisher’s exact test. Continuous outcomes for independent samples were analyzed by a Mann–Whitney-U test. To assess the influence of different continuous parameters on survival rate, we performed a median split followed by a Kaplan-Meier curve and a log-rank test. Ferritin was split by clinical cut offs into normal and high (women >140 ng/mL = high, men >160 ng/mL = high). NTproBNP was also split by clinical cut offs (women >150 mg/mL = high, men >100 mg/mL = high). A multivariate Cox regression analyzed the risk potential of several biomarkers for COVID-19 related death within the frame of a 90-day period. For the immune and coagulation system one parameter was chosen, respectively, as a representative using content-related and statistical criteria. Assumption of proportional hazards was checked graphically before the variables were entered into the model. Significant predictive biomarkers were analyzed by ROC. We determined optimal cut off values by Youden index. Sensitivity and specificity including 95% confidence intervals were estimated. Due to multiple testing, we used a Bonferroni correction: *p*-values < 0.004 were considered significant. Statistical analyses were performed using SPSS statistical software (version 26.0; IBM Corp, Armonk, NY, USA) and SAS software (version 9.4; SAS Institute, Inc., Cary, NC, USA).

## 3. Results

Table 1 shows baseline characteristics of the COVID-19 patients. A difference in age exists between the Exitus and Recovery group. People who survive COVID-19 are younger on average (*p* < 0.001, Mann-Whitney-*U* test). No significant difference was found in survival regarding sex (*p* = 0.855, chi^2^ test).

Table 2 shows levels of biomarkers at the time of the first visit of the investigated COVID-19 patients. The patients who recovered from COVID-19 had significantly lower plasma levels of KYN, CRP, IL-6, ferritin, NTproBNP, cTnT, and creatinin.

**Figure 1:**
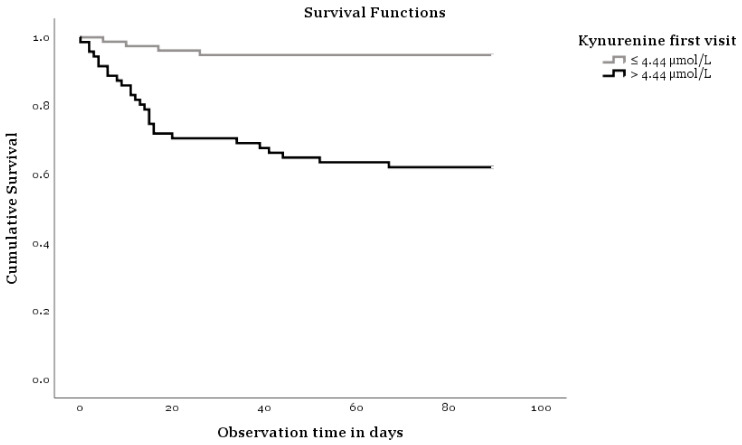
Kaplan-Meier analysis.

Figure 1 shows a Kaplan Meier analysis indicating that KYN concentrations influence the survival of COVID-19. The two curves of Figure 1 show differences concerning survival time and KYN levels measured on the first visit (log-rank test *p* < 0.001). Age, ferritin, and NTproBNP plasma concentrations also show clearly different trends (plots not shown). Thus, elderly patients have a lower survival rate compared to younger ones, and patients with clinically increased ferritin and NTproBNP concentrations have a lower survival rate compared to those with normal levels of these biomarkers. The log-rank test *p* values for age are <0.001, for ferritin 0.001, and for NTproBNP 0.001.

Table 3 shows the results of a Cox-Regression analysis. We entered several biomarkers into this model to assess their influence on the course of the disease. KYN showed a significant influence, for each µmol/L KYN more, the hazard ratio for dying from COVID-19 rises 1.19 (95% CI: 1.07–1.32). Age showed a tendency towards a significant influence, i.e., with each year of one’s life more, the hazard ratio of dying from COVID-19 grows 1.04 (95% CI: 1.01–1.07).

**Figure 2:**
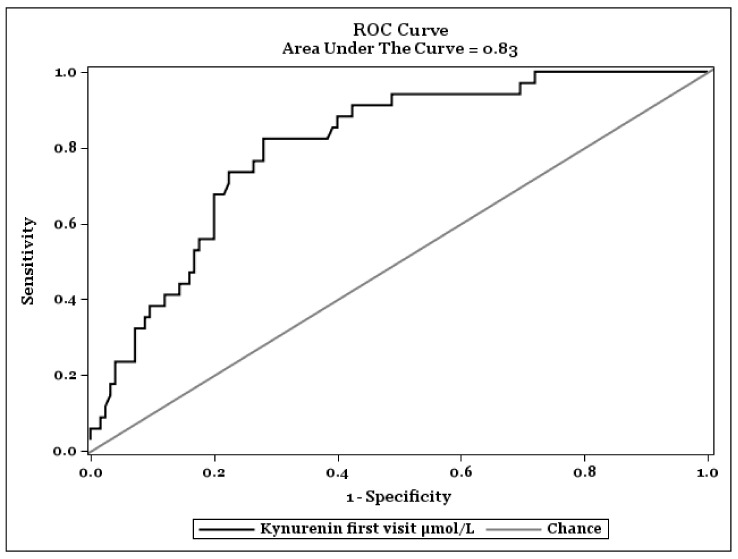
ROC Curve to find an optimal cut-off value of Kynurenine on progression of COVID-19.

Figure 2 shows a ROC-Analysis to find an optimal cut-off value of KYN plasma concentrations on fatal outcome of COVID-19. The optimal cut off value was specified by Youden index. The highest value of the Youden index was found at KYN concentrations of 4.82 nmol/L and had a sensitivity of 0.82 (95% CI: 0.66–0.95) and a specificity of 0.72 (95% CI: 0.65–0.82) in recognizing severity of the COVID-19 infection.

**Figure 3:**
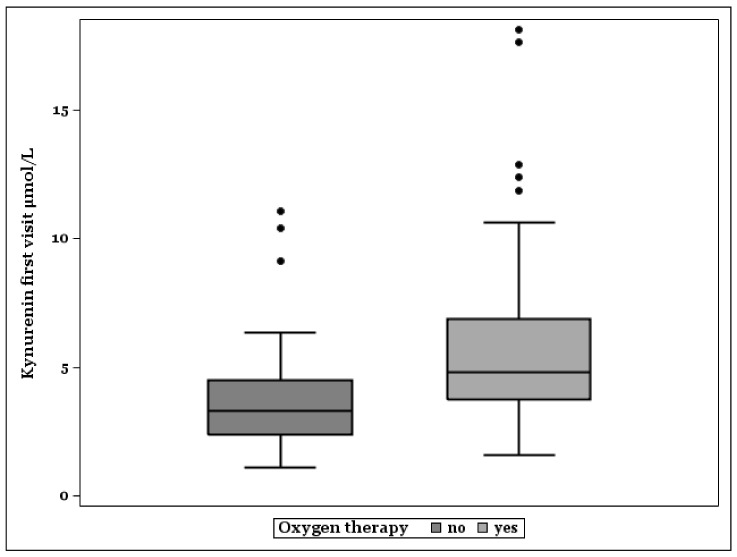
Box plots of kynurenine plasma levels in patients without, and with need of oxygen therapy.

Figure 3 shows that median KYN plasma levels were higher in patients treated with oxygen because of pulmonic manifestation of COVID-19 (N = 88). Non-survivors with oxygen therapy (N = 28) had the highest KYN concentrations (Median, Minimum–Maximum: 6.08, 3.14–18.12). Survivors with oxygen need showed higher KYN levels (N = 60, Median, Minimum–Maximum: 4.27, 1.58–12.87) than survivors without oxygen therapy (N = 56, Median, Minimum–Maximum: 3.23, 1.1–11.06).

## 4. Discussion

The present study seeks biomarkers that indicate a fatal outcome of COVID-19 in early phases of the infection when clinical symptoms are starting. To have such biomarkers in clinical routine can help save lives with a timely adjustment of therapy (e.g., application of dexamethasone) [29,30].

A reduced innate antiviral defense reaction coupled with exuberant inflammatory cytokine production is a driving feature of severe COVID-19 [31,32,33]. The life-threatening phase of severe COVID-19 does not begin immediately after diagnosis. Early biomarkers that indicate a shift to a fatal course are widely missing.

Metabolomics in COVID-19 patients proofed the existence of a characteristic immune-metabolic signature [34,35,36] with marked alterations in nitrogen metabolism, amino acid homeostasis, catabolism, transamination, and carbon metabolism affecting glucose and free fatty acids levels [34]. The metabolites in these affected pathways correlated with the increased IL-6 and CRP concentrations [34].

The investigations of the immune-metabolic signature also found signs of an activated TRP degradation pathway (TRP → Formyl-KYN → KYN) [35,37]. This pathway is determined by the enzymatic activity of indoleamine 2,3-dioxygenase 1 (IDO1), a pleotropic enzyme with immunoregulatory effects. IDO1 is physiologically expressed in various tissues such as lungs, small intestine, female genital tract, and placenta [38]. IDO is one of three enzymes that catalyze the first and rate-limiting step in the KYN pathway that is the O_2_-dependent oxidation of L-TRP to N-formyl-KYN. The other two enzymes are indoleamine-2,3-dioxygenase 2 (IDO2) [39] and tryptophan 2,3-dioxygenase (TDO) [40].

IDO1 is an immune checkpoint molecule [41] and acts as a negative regulator of inflammation and immune activation [42,43]. It limits T-cell function and starts mechanisms of immune tolerance, a function with potentially disastrous consequences in certain malignant diseases [44]. The immune modulating effects of IDO1 may also be of importance in infectious diseases like COVID-19.

Relevant for COVID-19, human airway epithelial cells abundantly express IDO [45]. At this anatomical site, IDO activation can limit inflammation in cases of severe pulmonic manifestations. Accordingly, deletion of IDO severely exacerbated inflammatory lung pathology in mice [46].

Furthermore, IDO induced TRP catabolites activate the aryl hydrocarbon receptor (AhR) [47]. The AhR is activated in SARS-CoV-2 infection where it impacts anti-viral immunity and lung basal cells involved in tissue repair [48].

Taking into account these functions, the IDO mediated dynamics of the TRP → KYN pathway are probably beneficial for the clinical course of COVID-19 pulmonic disease [45]. Furthermore, the TRP metabolism also interferes with oxidative stress, superoxide and NO production. Both, SARS-CoV-2 pneumonia and SARS-CoV-2 cytokine storm induce severe oxidative stress. Activated IDO1 may represent a local antioxidant defense against this [49].

Nevertheless, the immunosuppressive effects of IDO1 may outperform all positive effects [45], by exponentiation of an insufficient type I interferon production by the innate immune system in the early phase of severe COVID-19 [50,51,52,53]. Thus, SARS-CoV-2 can get out of control of the immune surveillance, a process that can result in a fatal hyperactive inflammation with cytokine storm and multi-organ failure. Moreover, SARS-CoV-2 spike protein induces ROS generation inducing oxidative stress [54]. KYN pathway metabolites increase these oxidative stress effects of SARS-CoV-2 in severe COVID-19 [20]. Probably these metabolites also play a role in post-COVID-19 syndrome [55].

These facts suggest that the stimulation of KYN pathway in COVID-19 is a double-edged sword. [56]. It may further explain the worse clinical courses of COVID-19 seen in patients with cancer and cardiovascular diseases where KYN concentrations are already high before the infection with SARS-CoV-2 (Mangge, Biomedicines, 2021, in press).

Analytically, the KYN/TRP ratio is an approximate measure of the IDO activity. However, this ratio depends on metabolism and its effects on half-lives of KYN and TRP. Unfortunately, in clinical studies, to which our investigation belongs, this ratio is not very reliable, being caused by a varying administration of TRP contenting parenteral infusions. Thus, we concentrate herein to KYN, the major degradant of TRP.

Our results showing KYN as a strong indicator for a deadly course of COVID-19 in a Kaplan-Meier analysis fit well into these observations. Interestingly our patients showed no significant difference in survival regarding sex as seen in other reports [57]. The patients who recovered from COVID-19 had significantly lower plasma levels of KYN, CRP, IL-6, ferritin, NTproBNP, cTnT, and creatinine. Verified in a Cox regression analysis, although weaker than KYN, age, ferritin, and NTproBNP also showed clearly different trends. Thus, elderly patients had a lower survival rate compared to younger ones, and patients with clinically increased ferritin and NTproBNP have a lower survival rate compared to those with normal levels of these biomarkers. For KYN, the hazard ratio for dying from COVID-19 rises 19% (95% CI: 7–32%) for each µmol/L increase of the plasma KYN value. For each year more of one’s life, the hazard ratio of dying from COVID-19 grows 4% (95% CI: 1–7%). By ROC-Analysis the optimal cut-off value of KYN for a fatal outcome of COVID-19 was found at KYN levels of 4.82 nmol/L with a sensitivity of 0.82 (95% CI: 0.66–0.95) and a specificity of 0.72 (95% CI: 0.65–0.82) in recognizing severity of the COVID-19 infection.

The KYN/TRP ratio was entered into the same Cox-model as shown above instead of KYN and showed the highest hazard ratio (13.25 CI: 1.89–92.75). However, some COVID-19 patients received TRP containing infusions. Despite the fact that the statistical significance was further improved, this substitution may cause an incalculable influence, and because the confidence interval was very broad, we did not include the KYN/TRP data in our final interpretation of results.

Accordingly, to the above-mentioned expression of IDO on the airway epithelial cells, the median KYN plasma levels were higher in patients treated with oxygen because of pulmonic manifestation of COVID-19. Non-survivors with oxygen therapy had the highest KYN levels and survivors without oxygen therapy demonstrated the lowest. These data underline the involvement of the KYN pathway in COVID-19 associated pulmonic disease. Interestingly, the increased KYN plasma concentrations antecede the clinically full expressed pulmonic symptoms because they are present at the time of admission. It is not possible to draw a conclusion from these data on whether KYN is more of an indicator of a protective mechanism at the starting point of pulmonic disease, or a pathfinder to a severe course of COVID-19 referring to immunosuppressive action and oxidative stress perturbation effects of IDO1. In addition, a possible role of IDO2 [4] remains to be investigated. 

Taken together, a tight association and a potential regulatory crosstalk between severe courses of COVID-19 and TRP metabolism exists [34]. This may be the best explanation now for the prognostic potency of KYN for a later fatal outcome of COVID-19.

A major limitation of our study is the relatively low number of investigated patients. Hence, we cannot answer the question to which extent preexisting cancer and/or cardiovascular diseases contribute to the increased KYN levels. Ongoing investigations will clarify this important point. Independently from this, KYN may be an interesting biomarker from the clinical point of view.

## 5. Conclusions

Kynurenine plasma concentrations may be a promising biomarker to predict an increased risk of mortality in SARS-CoV-2 infected people.

## Figures and Tables

**Table 1:** Baseline characteristics of COVID-19 patients.

	Exitus (*n* = 31)	Recovery (*n* = 117)
age (years)	80.0 (52.0, 98.0)	57.0 (18.0, 93.0)
Sex female	14 (45.2%)	55 (47.0%)
Sex male	17 (54.8%)	62 (53.0%)
ICU admission	13 (41.9%)	22 (18.8%)
normal ward	18 (58.1%)	95 (81.2%)
renal disease no	15 (48.4%)	98 (83.8%)
renal disease yes	16 (51.6%)	19 (16.2%)
CAD no	15 (48.4%)	93 (79.5%)
CAD yes	16 (51.6%)	24 (20.5%)
preexisting disease no	3 (9.7%)	51 (43.6%)
preexisting disease yes	28 (90.3%)	66 (56.4%)
cancer no	24 (80.0%)	106 (91.4%)
cancer yes	6 (20.0%)	10 (8.6%)
hypertension	8 (25.8%)	71 (60.7%)
oxygen therapy	28 (90.3%)	60 (51.7%)
pulmonary disease no	25 (80.6%)	101 (87.1%)
pulmonary disease yes	6 (19.4%)	15 (12.9%)

Age displayed as median (minimum, maximum); all other variables displayed as N (%).

**Table 2:** Levels of biomarkers found between the groups.

Plasma Value 1st Visit	N	Exitus Median (min, max)	N	Recovery Median (min, max)	*p*-Value
Kynurenine, µmol/L	31	6.1 (3.1, 18.1)	117	3.7 (1.1, 12.9)	<0.001
CRP, mg/dL	31	87.0 (3.8, 315.3)	117	22.0 (0.6, 336.9)	<0.001
Interleukin-6, pg/mL	31	79.9 (7.5, 614.0)	117	18.8 (1.5, 3086.0)	<0.001
Ferritin, ng/mL	31	619.0 (78.0, 23,706.0)	117	336.0 (7.0, 14,553.0)	0.008
NTproBNP, pg/mL	31	2110.0 (21.0, 70,000.0)	117	125.0 (5.0, 70,000.0)	<0.001
cTnT, pg/mL	31	51.0 (3.0, 851.0)	117	7.0 (3.0, 1020.0)	<0.001
Creatinin, mg/dL	31	1.4 (0.6, 13.4)	117	1.0 (0.5, 8.4)	<0.001
D-Dimer, mg/L	21	1.8 (0.2, 33.0)	91	0.7 (0.2, 33.0)	-*-

Min = minimum, max = maximum; *p*-value estimated by a Mann-Whitney-*U* test; * not tested due to incomplete data.

**Table 3:** Results of the Cox-Regression.

Parameter	Category	Hazard Ratio	Lower 95% Confidence Limit	Upper 95% Confidence Limit	*p*-Value
Sex	male versus female	1.03	0.49	2.18	0.938
Age, years	-	1.04	1.01	1.07	0.008
Kynurenine, µmol/L	-	1.19	1.07	1.32	0.001
Ferritin, ng/mL grouped	normal versus high	1.71	0.51	5.73	0.389
NTproBNP, pg/mL grouped	normal versus high	6.78	0.82	55.90	0.075

Reference categories for categorical parameters sex = male; Ferritin = normal; BNP = normal.

## Data Availability

Data is contained within the article.
